# Preparation and Characterization of Latex Particles as Potential Physical Shale Stabilizer in Water-Based Drilling Fluids

**DOI:** 10.1155/2014/895678

**Published:** 2014-06-23

**Authors:** Junyi Liu, Zhengsong Qiu, Wei'an Huang, Dingding Song, Dan Bao

**Affiliations:** College of Petroleum Engineering, China University of Petroleum, Qingdao 266580, China

## Abstract

The poly(styrene-methyl methacrylate) latex particles as potential physical shale stabilizer were successfully synthesized with potassium persulfate as an initiator in isopropanol-water medium. The synthesized latex particles were characterized by Fourier transform infrared spectroscopy (FT-IR), particle size distribution measurement (PSD), transmission electron microscopy (TEM), and thermal gravimetric analysis (TGA). FT-IR and TGA analysis confirmed that the latex particles were prepared by polymerization of styrene and methyl methacrylate and maintained good thermal stability. TEM and PSD analysis indicated that the spherical latex particles possessed unimodal distribution from 80 nm to 345 nm with the D90 value of 276 nm. The factors influencing particle size distribution (PSD) of latex particles were also discussed in detail. The interaction between latex particles and natural shale cores was investigated quantitatively via pore pressure transmission tests. The results indicated that the latex particles as potential physical shale stabilizer could be deformable to bridge and seal the nanopores and microfractures of shale to reduce the shale permeability and prevent pore pressure transmission. What is more, the latex particles as potential physical shale stabilizer work synergistically with chemical shale stabilizer to impart superior shale stability.

## 1. Introduction

In the past decade, shale gas, as a clean and unconventional energy, has become progressively important in the energy landscape worldwide [1–5]. In drilling engineering, wellbore instability in shale, such as hole collapse, tight hole, and lost circulation, has still been a challenge especially in horizontal drilling process due to fluid penetration of water-based drilling fluids into shale matrix and subsequent pore pressure build-up and sloughing of the wellbore [6–8]. Generally, oil-based drilling fluids were chosen to drill in shale formation because of no chemical interaction between oil and shale [9]. However, the increasingly stringent environmental and economic requirements restricted its wide use and specially designed water-based drilling fluid would be an alternative. Maintaining wellbore stability using water-based drilling fluid could be achieved by sealing/consolidating wellbore physically or chemically to prevent pore pressure transmission [10, 11]. Recently, many solutions have been proposed for plugging the formation through different mechanisms, such as calcium carbonate, asphaltenes, polyglycols, and polymers, but only marginal success has been achieved [12, 13]. Geologically, gas shales, a sedimentary rock, are mainly composed of clay-sized particles and are believed to be the low porosity and ultralow permeability reservoir with a significant pore volume in the nanopore range [14–16]. But conventional particles are too large to bridge and seal nanoscale pore throats and microfractures of shale and novel plugging agents in nanoscale are needed for shale stability.

Historically, emulsion polymerization was widely used in the preparation of polymer materials, especially nanomaterials, but the water resistance and surface smoothness of polymers were influenced inevitably by residual emulsifiers [17, 18]. The emulsifier-free emulsion polymerization emerged at the right moment [19, 20], and polymers could be synthesized without emulsifiers or just with small amount under critical micelle concentration (CMC). But it was too difficult to control the particles size and improve the conversion rate and stability of emulsifier-free latex [21]. Recently, solvothermal method has been applied to emulsifier-free polymerization [22], and high reacting temperature and reacting pressure could make it possible to decrease particle size and improve the stability of emulsifier-free latex.

In this work, the emulsifier-free latex particles as potential physical shale stabilizer in water-based drilling fluids were synthesized by emulsifier-free emulsion polymerization of styrene (St) and methyl methacrylate (MMA) using solvothermal method with potassium persulfate as an initiator in isopropanol-water medium. The newly synthesized latex particles were characterized in detail and the influencing factors on particle size distribution (PSD) of latex particles, such as isopropanol volume, reacting temperature, and initiator concentration, were also discussed. Moreover, the interaction between latex particles and natural shale cores was investigated quantitatively via pore pressure transmission tests.

## 2. Experimental Materials and Methods

### 2.1. Materials

The monomers of styrene (St) and methyl methacrylate (MMA), obtained from Sinopharm Chemical Reagent Co. Ltd. (China), were distilled under vacuum before use. The initiator, potassium persulfate, was obtained from Aladdin Reagent Co. Ltd. (China) and was recrystallized for purification. Isopropanol and sodium chloride, AR grade, were obtained from Sinopharm Chemical Reagent Co. Ltd. (China) and used as received. The distilled water was used throughout the experiments.

The chemical shale stabilizer, SDCS, is an aluminum complex self-developed to impart shale stability. The aluminum complex could completely dissolve in the water-based drilling fluids when pH of the fluids is maintained above 11. When interacting with formation water of low pH, it could precipitate in the shale matrix and decrease the shale permeability, thus providing a barrier to pore pressure transmission. In addition, the chemical shale stabilizer could withstand high temperature and salt concentration.

The shale samples, here, were obtained from the Sichuan Basin, China. The main clay minerals of shale samples were determined to be illite/smectite and illite by X-ray diffraction analysis (Table 1), with cation exchange capacity and surface area of 40 mmol/g and 49.65 m^2^/g, respectively. The cylindrical shale cores were used in the pore pressure transmission tests with a diameter of 2.54 cm and a length of 0.80 cm.

### 2.2. Preparation of P(St-MMA) Latex Particles

The P(St-MMA) latex particles were prepared by the emulsifier-free polymerization of styrene and methyl methacrylate using solvothermal method with potassium persulfate as an initiator in isopropanol-water medium. The styrene, methyl methacrylate, potassium persulfate, isopropanol, and distilled water of characteristic concentration were successively added into a hydrothermal synthesis reaction kettle. Then, the mixture above was stirred using electromagnetic stirring at room temperature for 15 min and at 90°C for another 2.5 h. The P(St-MMA) latex particles with different reaction conditions were prepared using similar methods. The recipes for synthesis of P(St-MMA) latex particles were shown in Table 2.

### 2.3. Characterization of P(St-MMA) Latex Particles

The FT-IR spectra were acquired by using NEXUS 670 FT-IR spectrometer (Thermo Nicolet, USA), scanning from 4000 to 400 cm^−1^. The purified latex particles were dried under vacuum at 80°C, and mixture of latex particles samples and potassium bromide (KBr) was pressed into pellets for FT-IR analysis. The transmission electron microscopy (TEM) images of latex particles were taken with JEOL JEM-2100UHR TEM using an accelerating voltage of 200 kV. The particle size distribution (PSD) of P(St-MMA) latex particles was analyzed with dynamic light scattering using Mastersizer 3000 (Malvern, UK). The samples of TEM and PSD were diluted before testing. The thermal gravimetric analysis (TGA) was conducted with a simultaneous thermal analyzer (NETZSCH, Germany) at a heating rate of 0~50°C/min under nitrogen atmosphere. It should be pointed out that the latex particles used for characterization were synthesized using S-3 recipe (Table 2).

### 2.4. Pore Pressure Transmission Tests

**Figure 1:**
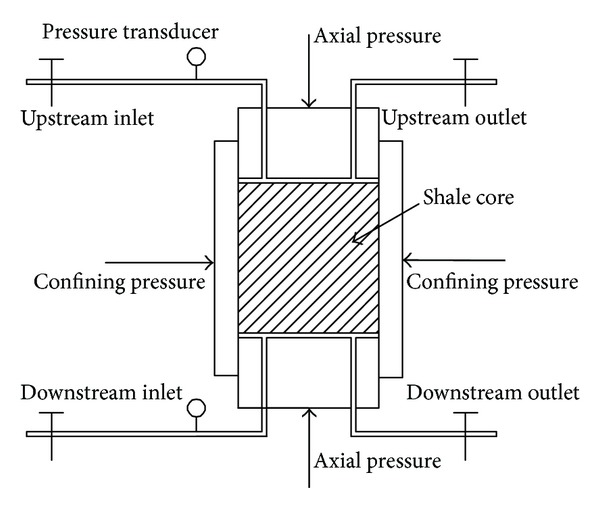
Schematic of pore pressure transmission test setup.

The pore pressure transmission test was developed using the pressure transmission technique to characterize the hydraulic properties of shale and the pore pressure transmission tests in this paper were performed on the simulation equipment for hydramechanics coupling of shale, developed by China University of Petroleum (East China) [23], and basic components of the simulation equipment are illustrated in Figure 1.

During the pore pressure transmission test, shale cores were subjected to hydraulic or osmotic gradients or both when exposed to upstream and downstream fluids. The confining pressure and axial pressure were all 5.0 MPa and upstream pressure was maintained at 3.0 MPa. The initial downstream pressure was 1.0 MPa. The pore pressure transmission tests were performed at 70°C. The downstream pressure, namely, pore pressure, was monitored throughout the tests. With no chemical potential difference between upstream and downstream fluids, the downstream pressure would become equal to the applied constant fluids pressure at the upstream because of nonzero permeability of shale cores. But, when there is chemical potential difference present, any difference between the downstream pressure and the applied constant fluid pressure could be measured in response to osmotic pressure.

The permeability of shale cores could be calculated using formula (1) [24]. Consider

(1) K=μβVLA  ×(ln⁡(Pm−P0Pm−P(L,t2))−ln⁡(Pm−P0Pm−P(L,t1)))×(t2−t1)−1,

where *K* is the permeability of shale cores, *μ*m^2^; *μ* is the viscosity of fluids, mPa.s; *β* is the static compression ratio of fluids, MPa^−1^; *V* is the enclosed volume of downstream fluids, cm^3^; *L* is the length of shale cores, cm; *A* is the cross-sectional area, cm^2^; *t* is total experimental time, s; *P*_*m*_ is the upstream pressure, MPa; *P*_0_ is the pore pressure, MPa; *P*(*L*, *t*) is the real-time downstream pressure, MPa.

Shale could act as a nonideal semipermeable membrane, and membrane efficiency was defined as the ratio of actual osmotic pressure and ideal osmotic pressure to characterize its nonideality [25]. Consider

(2) $$\begin{array}{c} \sigma =\frac{\Delta P}{\Delta \Pi }=\frac{\Delta P}{\left(RT/V_{W}\right)\ln\left(a_{w}^{\text{sh}}/a_{w}^{\text{df}}\right)}, \end{array}$$

where *σ* is the membrane efficiency of shale cores, %; Δ*P* is the actual osmotic pressure, MPa; *R* is the ideal gas constant, 8.314 J*·*mol^−1^ *·*K^−1^; *T* is the test temperature, K; *V*_*W*_ is the partial molar volume of water, 18 cm^3^ *·*mol^−1^; *a*_*w*_^sh^ is the water activity of pore water; *a*_*w*_^df^ is the water activity of drilling fluids.

In addition, the pore structure of shale cores before and after tests was characterized by Hitachi S-4800 field-emission scanning electron microscope (SEM) analysis.

## 3. Results and Discussion

### 3.1. Characterization of P(St-MMA) Latex Particles

#### 3.1.1. FT-IR Analysis

**Figure 2:**
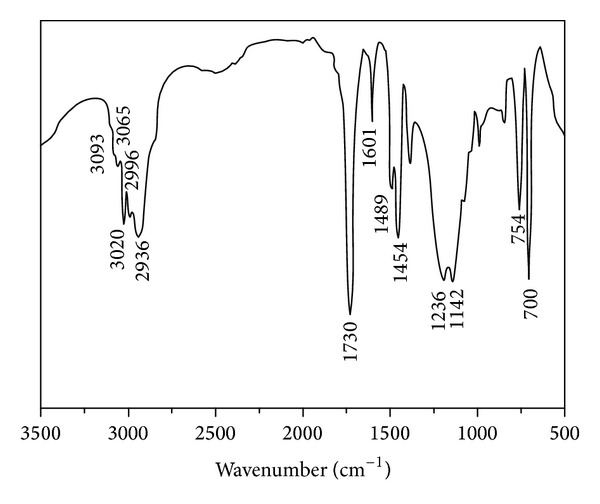
FT-IR spectrum of purified latex particles.

The FT-IR spectrum of purified latex particles (Figure 2) showed absorption peak at around 1730 cm^−1^, corresponding to the C=O stretching band, and two characteristic absorption peaks at around 1236 cm^−1^ and 1142 cm^−1^, corresponding to the C–O–C symmetric stretching band. The stretching vibrations of benzene skeleton were presented at around 1601 cm^−1^, 1489 cm^−1^, and 1454 cm^−1^, and 754 cm^−1^ and 700 cm^−1^ were characteristic bending vibrations of single substitution benzene ring. It was also observed from Figure 2 that the C–H absorption peaks of single substitution benzene ring were found at 3093 cm^−1^, 3065 cm^−1^, 3020 cm^−1^, and 2996 cm^−1^ [26, 27]. Because the P(St) and P(MMA) have been separated before testing, the above discussion confirmed that the newly synthesized latex particles were copolymers of St and MMA.

#### 3.1.2. TGA Analysis

**Figure 3:**
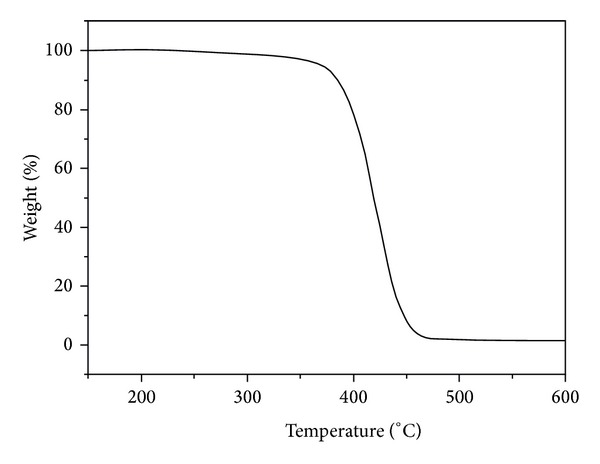
TGA curve of purified latex particles.

The weight loss observed up to 200°C was attributed to the desorption of physically absorbed water and dehydration of the hydrated cations. The organic compounds were decomposed between 200 and 500°C [28]. From the TGA curve (Figure 3), the purified latex particles began to decompose at around 250°C and the weight loss of latex particles would not increase significantly until temperature increased up to 380°C, indicating that the newly synthesized latex particles maintain good thermal stability. This can be attributed to the fact that the rigidity of P(St-MMA) is enhanced owing to the introduction of benzene ring [29].

#### 3.1.3. PSD and TEM Analysis

**Figure 4:**
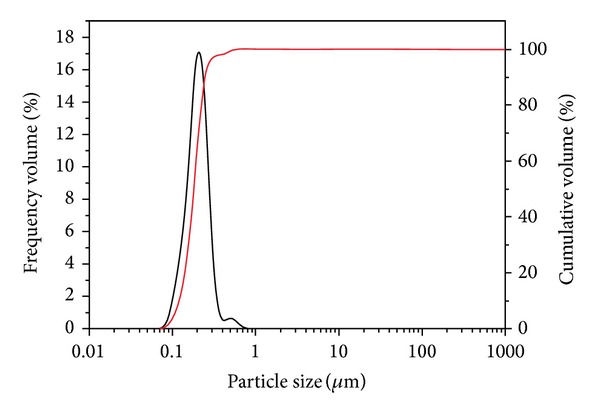
Particle size distribution of latex particles.

**Figure 5:**
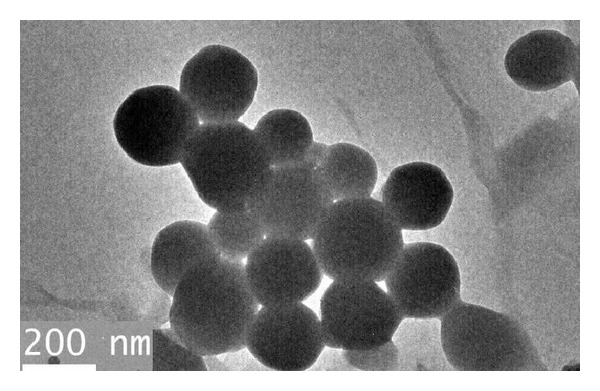
TEM micrograph of latex particles.

As can be seen from Figure 4, the particle size of latex particles almost unimodally distributed from 80 nm to 345 nm with the D90 value of 276 nm in addition to some aggregates with larger sizes. After entering pore throats and microfractures of shale, the coarse particles are prone to bridge or seal the largest openings of shale formation and finer particles are necessary to fill the voids between coarse particles to produce a tight immobile plug [30].  Figure 5 displays the representative TEM micrograph of P(St-MMA) latex particles. It can be seen that the latex particles are spherical and the average particle size determined by TEM is about 200 nm, which is consistent with the D50 value (208 nm) of PSD analysis.

### 3.2. Factors Influencing PSD of P(St-MMA) Latex Particles

When it comes to reducing shale pore pressure transmission, particle size distribution is one of the most important factors influencing plugging mechanism and efficiency of pore throats and microfractures [31, 32], and D90, cumulative amount of 90% of particles which are smaller than the size, is commonly defined as a characteristic parameter. Thus, the effects of isopropanol volume, reacting temperature, and initiator concentration on particle size distribution (PSD) of latex particles were discussed here.

#### 3.2.1. Isopropanol Volume

**Figure 6:**
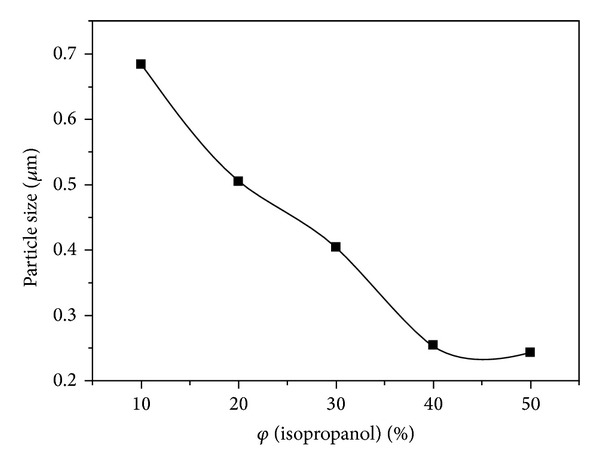
Effect of isopropanol volume on D90.

It can be seen from Figure 6 that the D90 value of latex particles decreased continuously with the increase of isopropanol volume from 10% to 50%. This can be attributed to the fact that, owing to the introduction of isopropanol, the reacting medium has lower polarity and surface tension, and it is beneficial to dispersion of droplets or particles. Additionally, isopropanol acts as chain transfer agent in the polymerization, and it could generate more surface-active oligomers in the nucleation stage and prevent particles aggregation, thus decreasing the latex particle size [33].

#### 3.2.2. Reacting Temperature

**Figure 7:**
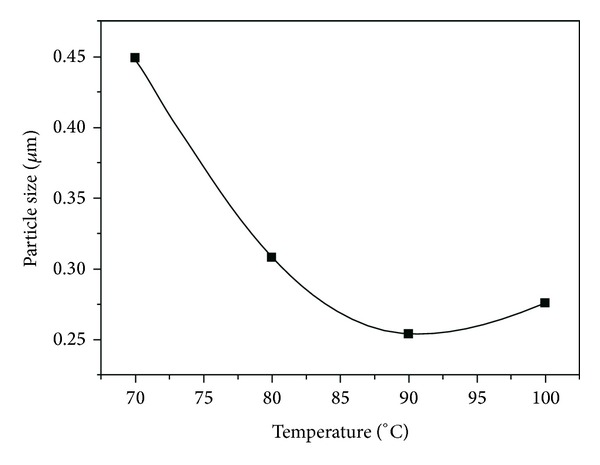
Effect of reacting temperature on D90.

The effect of reacting temperature on D90 value of latex particles is shown in Figure 7. It can be seen that the D90 value of latex particles decreased with the increase of reacting temperature, but when reacting temperature exceeded 90°C, the D90 value began to increase with the increase of reacting temperature. This can be attributed to two aspects. On the one hand, high reacting temperature increases the solubility of monomers and decomposition rate of initiators and generates more latex particles in the nucleation stage, thus decreasing the latex particle size. On the other hand, high reacting temperature could also intensify the Brownian motion and deteriorate dispersion stability of latex particles. Additionally, due to hydrophobic hydration effect and pressure effect on solubility of hydrophobic monomers, the increase of reacting temperature would also enhance the coalescence of oligomer micelle, thus increasing the latex particle size [34, 35].

#### 3.2.3. Initiator Concentration

**Figure 8:**
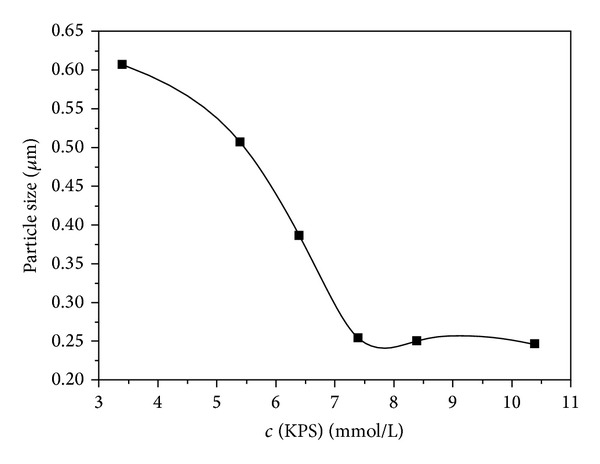
Effect of initiator concentration on D90.

Figure 8 depicts the effect of initiator concentration on D90 value of latex particles. It can be seen that the D90 value of latex particles decreased with the increase of initiator concentration, and when the initiator concentration exceeded 7.4 mmol/L, the D90 value would not decrease any longer. The increase of initiator concentration makes it beneficial to generate more negatively charged surface-active oligomer micelles that are absorbed on latex particles, thus preventing latex particles aggregation and decreasing latex particles size. The high initiator concentration could also accelerate nucleation rate and polymerization rate because of high free radical production.

### 3.3. Pore Pressure Transmission Tests

During the pore pressure transmission tests, the P(St-MMA) latex particles were used as physical shale stabilizer (SDPS) and self-developed aluminum complex was used as chemical shale stabilizer (SDCS). The downstream fluid was 3% sodium chloride solution (DSFL, *a*_*w*_ = 0.998), and the upstream fluids were 3% sodium chloride solution (USFL-1, *a*_*w*_ = 0.998), 20% sodium chloride solution (USFL-2, *a*_*w*_ = 0.875), 3% sodium chloride solution with 3% SDPS (USFL-3, *a*_*w*_ = 0.998), 20% sodium chloride solution with 2% SDPS, and 1% SDCS (USFL-4, *a*_*w*_ = 0.875), respectively.

**Figure 9:**
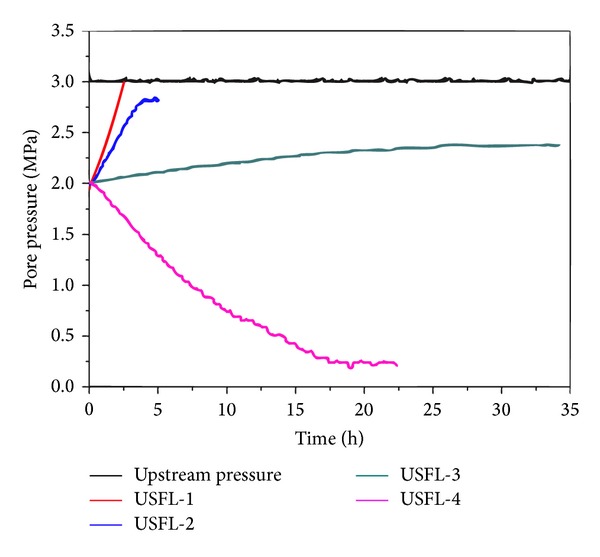
Pore pressure transmission test curves.

The curves of pore pressure transmission tests are shown in Figure 9. It can be concluded that it takes more time for pore pressure build-up under hydraulic pressure when interacting with USFL-3. The permeability of shale cores could be calculated according to formula (1). After interacting with USFL-3, the permeability decreased significantly from 1.26 × 10^−7^ *μ*m^2^ (USFL-1) to 1.01 × 10^−8^ *μ*m^2^. Because there is no chemical potential difference between USFL-1/USFL-3 and DSFL, the effect of preventing or reducing pore pressure transmission is mainly related to internally bridging and sealing shale microfractures of deformable P(St-MMA) latex particles.

When the upstream fluid was changed to 20% sodium chloride solution (USFL-2, *a*_*w*_ = 0.875), the pore pressure (downstream pressure) would be less than upstream pressure due to chemical potential difference between USFL-2 and DSFL. After interacting with USDL-4, the downstream pressure decreased gradually and offset the hydraulic pressure. It is concluded that shale acts as a semipermeable membrane, and the hydraulic pressure could be offset by the backflow caused by the development of osmosis when there exists chemical potential difference. According to formula (2), the membrane efficiency of natural shale core is only 1.61%, and it increases to 14.71% after interacting with USDL-4.

**Figure 10:**
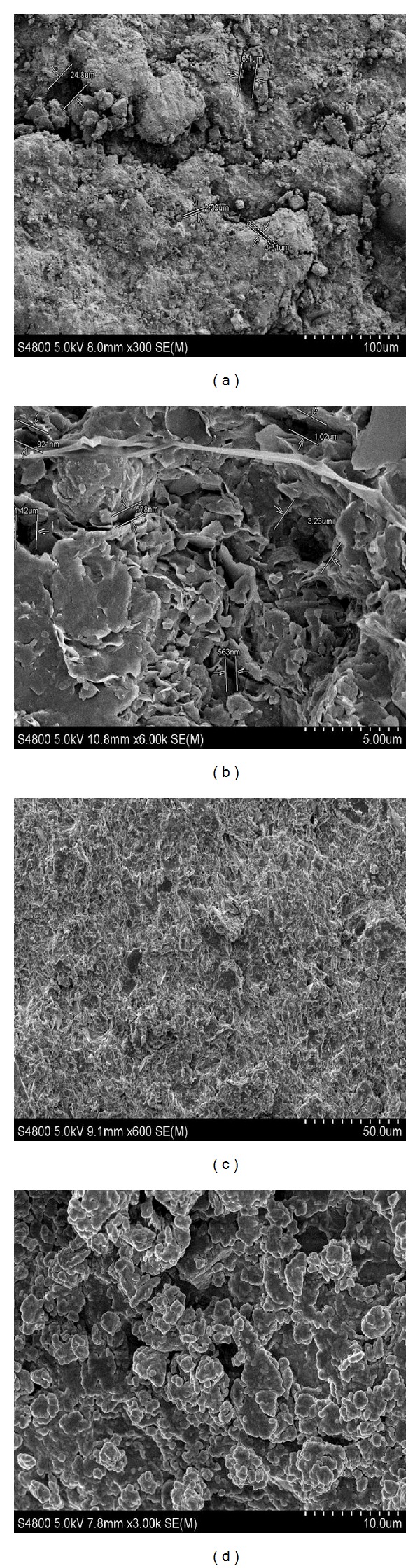
SEM photographs of shale cores: (a)-(b) natural shale cores; (c)-(d) shale cores after interacting with USFL-4.

As shown in Figure 10, the P(St-MMA) latex particles could be deformable to internally bridge and seal pore throats and microfractures of shale cores, and the aluminum complex would precipitate when the drilling fluid filtrate is exposed to formation water and the precipitation could block and seal pore throats and microfractures [36]. What is more, the P(St-MMA) latex particles and aluminum complex could act synergistically to reduce pore pressure transmission and increase membrane efficiency of shale cores, thus improving shale stability in drilling engineering.

## 4. Conclusions

The P(St-MMA) latex particles have been successfully prepared by emulsifier-free emulsion polymerization of St and MMA, using potassium persulfate as an initiator in isopropanol-water medium. The latex particles possessed unimodal distribution from 80 nm to 345 nm with the D90 value of 276 nm, and the particle size was influenced significantly by isopropanol volume, reacting temperature, and initiator concentration. The latex particles could disperse uniformly in the water-based drilling fluids as potential physical shale stabilizer and they could be deformable to internally bridge and seal pore throats and microfractures of shale. What is more, a cooperative action was observed in P(St-MMA) latex particles (physical shale stabilizer) and aluminum complex (chemical shale stabilizer) to reduce pore pressure transmission and increase membrane efficiency of shale, thus improving shale wellbore stability. The newly synthesized latex particles were alternatives to conventional plugging agents in water-based drilling fluids for shale gas.

## Figures and Tables

**Table 1:** The mineralogical composition of shale samples.

X-ray diffraction	% weight
Quartz	57
K feldspar	4
Plagioclase	6
Siderite	4
Clay	29
Kaolinite	14
Chlorite	12
Illite	36
Illite/smectite	38

**Table 2:** Recipes of the P(St-MMA) latex particles.

Number	[St] : [MMA]	[KPS]/(mmol/L)	*V* (isopropanol) : *V* (water)	Temperature (°C)	Time (h)
S-1	1 : 1	7.40	2 : 3	70	2.5
S-2	1 : 1	7.40	2 : 3	80	2.5
S-3	1 : 1	7.40	2 : 3	90	2.5
S-4	1 : 1	7.40	2 : 3	100	2.5
S-5	1 : 1	7.40	2 : 3	90	2.0
S-6	1 : 1	7.40	2 : 3	90	3.0
S-7	1 : 1	7.40	2 : 3	90	3.5
S-8	1 : 1	7.40	2 : 3	90	4.0
S-9	1 : 1	7.40	1 : 4	90	2.5
S-10	1 : 1	7.40	3 : 7	90	2.5
S-11	1 : 1	7.40	1 : 1	90	2.5
S-13	1 : 1	5.40	2 : 3	90	2.5
S-14	1 : 1	6.40	2 : 3	90	2.5
S-15	1 : 1	10.40	2 : 3	90	2.5
